# Tris[4-(methyl­sulfan­yl)phen­yl]arsine

**DOI:** 10.1107/S160053681002876X

**Published:** 2010-07-24

**Authors:** Omar bin Shawkataly, Imthyaz Ahmed Khan, Chin Sing Yeap, Hoong-Kun Fun

**Affiliations:** a Chemical Sciences Programme, School of Distance Education, Universiti Sains Malaysia, 11800 USM, Penang, Malaysia; b X-ray Crystallography Unit, School of Physics, Universiti Sains Malaysia, 11800 USM, Penang, Malaysia

## Abstract

In the title compound, C_21_H_21_AsS_3_, the three benzene rings make dihedral angles of 88.41 (10), 87.75 (9) and 74.74 (10)° with each other. The methyl­sulfanyl groups are roughly coplanar with their attached benzene rings [C—S—C—C torsion angles = −7.6 (2), 11.2 (2) and 4.1 (2)°]. In the crystal, weak C—H⋯π inter­actions link the mol­ecules.

## Related literature

For related structures of tris­aryl­arsines with osmium and ruthenium, see: Cullen *et al.* (1995 ▶); Shawkataly *et al.* (2009*a* ▶,*b* ▶, 2010*a* ▶,*b* ▶). For the stability of the temperature controller used in the data collection, see: Cosier & Glazer (1986 ▶).
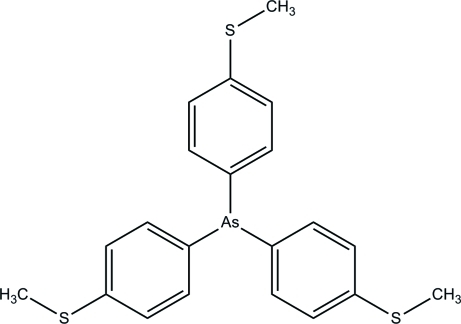


## Experimental

### 

#### Crystal data

C_21_H_21_AsS_3_
*M* *_r_* = 444.48
Monoclinic, 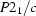

*a* = 11.0839 (2) Å
*b* = 11.4556 (2) Å
*c* = 17.3247 (2) Å
β = 110.860 (1)°
*V* = 2055.58 (6) Å^3^
*Z* = 4
Mo *K*α radiation
μ = 1.96 mm^−1^
*T* = 100 K
0.35 × 0.13 × 0.11 mm

#### Data collection

Bruker SMART APEXII CCD diffractometer
Absorption correction: multi-scan (*SADABS*; Bruker, 2009 ▶) *T*_min_ = 0.545, *T*_max_ = 0.821
31130 measured reflections
7111 independent reflections
5098 reflections with *I* > 2σ(*I*)
*R*_int_ = 0.051

#### Refinement

*R*[*F* ^2^ > 2σ(*F* ^2^)] = 0.039
*wR*(*F* ^2^) = 0.097
*S* = 1.01
7111 reflections
229 parameters
H-atom parameters constrained
Δρ_max_ = 0.86 e Å^−3^
Δρ_min_ = −0.51 e Å^−3^

### 

Data collection: *APEX2* (Bruker, 2009 ▶); cell refinement: *SAINT* (Bruker, 2009 ▶); data reduction: *SAINT*; program(s) used to solve structure: *SHELXTL* (Sheldrick, 2008 ▶); program(s) used to refine structure: *SHELXTL*; molecular graphics: *SHELXTL*; software used to prepare material for publication: *SHELXTL* and *PLATON* (Spek, 2009 ▶).

## Supplementary Material

Crystal structure: contains datablocks global, I. DOI: 10.1107/S160053681002876X/hb5559sup1.cif

Structure factors: contains datablocks I. DOI: 10.1107/S160053681002876X/hb5559Isup2.hkl

Additional supplementary materials:  crystallographic information; 3D view; checkCIF report

## Figures and Tables

**Table 1:** Hydrogen-bond geometry (Å, °) *Cg*1 is the centroid of the C7–C12 benzene ring.

*D*—H⋯*A*	*D*—H	H⋯*A*	*D*⋯*A*	*D*—H⋯*A*
C21—H21*A*⋯*Cg*1^i^	0.96	2.55	3.441 (3)	155

Symmetry code: (i) 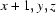.

**Fig. 1.**
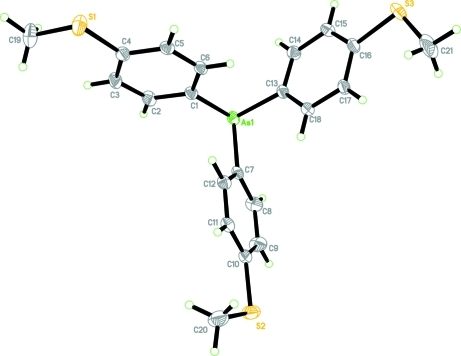
The molecular structure of (I) with 50% probability ellipsoids for non-H atoms.

**Fig. 2.**
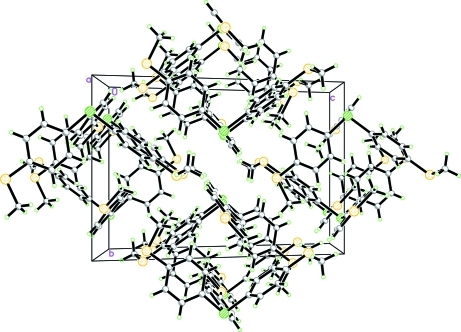
The crystal packing of (I), viewed down the a axis, showing the molecules are stacked along a axis.

## supplementary crystallographic information

### Comment

Trisarylarsines are used in the synthesis of osmium and ruthenium cluster derivatives (Cullen *et al.*, 1995; Shawkataly *et al.*, 2009*a*, *b*, 2010*a*, *b*).

The three benzene rings of the title compound (Fig. 1) make dihedral angles (C1–C6/C7–C12, C1–C6/C13–C18 and C7–C12/C13–C18) of 88.41 (10), 87.75 (9) and 74.74 (10)° with each other respectively. The methylsulfanyl groups are nearly coplanar with the attached benzene rings [torsion angles of C19–S1–C4–C3 = -7.6 (2), C20–S2–C10–C9 = 11.2 (2) and C21–S3–C16–C17 = 4.1 (2)°]. In the crystal structure, the molecules are stacked along *a* axis (Fig. 2). Weak intermolecular C—H···π interactions further stabilize the crystal structure (Table 1).

### Experimental

The reactions were conducted under an atmosphere of high purity nitrogen using standard Schlenk techniques and tetrahydrofuran (THF) dried over sodium metal. Tris(4-(methylsulfanyl)phenyl)arsine was prepared from arsenic trichloride and 4-(methylsulfanyl)phenylmagnesium bromide in tetrahydrofuran. Colourless blocks of (I) were obtained by slow evaporation from a chloroform solution.

### Refinement

All hydrogen atoms were positioned geometrically and refined using a riding model with C–H = 0.93 or 0.96 Å and *U*_iso_(H) = 1.2 or 1.5*U*_eq_(C). The rotating group model was applied to the methyl groups.

### Crystal data

**Table:**

C_21_H_21_AsS_3_	*F*(000) = 912
*M**_r_* = 444.48	*D*_x_ = 1.436 Mg m^−^^3^
Monoclinic, *P*2_1_/*c*	Mo *K*α radiation, λ = 0.71073 Å
Hall symbol: -P 2ybc	Cell parameters from 6720 reflections
*a* = 11.0839 (2) Å	θ = 2.7–31.0°
*b* = 11.4556 (2) Å	µ = 1.96 mm^−^^1^
*c* = 17.3247 (2) Å	*T* = 100 K
β = 110.860 (1)°	Block, colourless
*V* = 2055.58 (6) Å^3^	0.35 × 0.13 × 0.11 mm
*Z* = 4	

### Data collection

**Table:**

Bruker SMART APEXII CCD diffractometer	7111 independent reflections
Radiation source: fine-focus sealed tube	5098 reflections with *I* > 2σ(*I*)
graphite	*R*_int_ = 0.051
φ and ω scans	θ_max_ = 32.0°, θ_min_ = 2.0°
Absorption correction: multi-scan (*SADABS*; Bruker, 2009)	*h* = −16→16
*T*_min_ = 0.545, *T*_max_ = 0.821	*k* = −11→17
31130 measured reflections	*l* = −25→25

### Refinement

**Table:**

Refinement on *F*^2^	Primary atom site location: structure-invariant direct methods
Least-squares matrix: full	Secondary atom site location: difference Fourier map
*R*[*F*^2^ > 2σ(*F*^2^)] = 0.039	Hydrogen site location: inferred from neighbouring sites
*wR*(*F*^2^) = 0.097	H-atom parameters constrained
*S* = 1.00	*w* = 1/[σ^2^(*F*_o_^2^) + (0.0486*P*)^2^] where *P* = (*F*_o_^2^ + 2*F*_c_^2^)/3
7111 reflections	(Δ/σ)_max_ = 0.001
229 parameters	Δρ_max_ = 0.86 e Å^−^^3^
0 restraints	Δρ_min_ = −0.51 e Å^−^^3^

### Special details

**Table:**

Experimental. The crystal was placed in the cold stream of an Oxford Cryosystems Cobra open-flow nitrogen cryostat (Cosier & Glazer, 1986) operating at 100.0 (1) K.
Geometry. All e.s.d.'s (except the e.s.d. in the dihedral angle between two l.s. planes) are estimated using the full covariance matrix. The cell e.s.d.'s are taken into account individually in the estimation of e.s.d.'s in distances, angles and torsion angles; correlations between e.s.d.'s in cell parameters are only used when they are defined by crystal symmetry. An approximate (isotropic) treatment of cell e.s.d.'s is used for estimating e.s.d.'s involving l.s. planes.
Refinement. Refinement of *F*^2^ against ALL reflections. The weighted *R*-factor *wR* and goodness of fit *S* are based on *F*^2^, conventional *R*-factors *R* are based on *F*, with *F* set to zero for negative *F*^2^. The threshold expression of *F*^2^ > σ(*F*^2^) is used only for calculating *R*-factors(gt) *etc*. and is not relevant to the choice of reflections for refinement. *R*-factors based on *F*^2^ are statistically about twice as large as those based on *F*, and *R*- factors based on ALL data will be even larger.

### Fractional atomic coordinates and isotropic or equivalent isotropic displacement parameters (Å^2^)

**Table:**

	*x*	*y*	*z*	*U*_iso_*/*U*_eq_	
As1	0.851830 (19)	0.703422 (18)	0.496255 (12)	0.01802 (6)	
S1	0.60863 (5)	1.03859 (5)	0.17158 (3)	0.03019 (13)	
S2	0.83788 (6)	1.05708 (5)	0.78874 (3)	0.02996 (13)	
S3	1.44348 (5)	0.76333 (6)	0.50798 (4)	0.03192 (14)	
C1	0.78015 (19)	0.80668 (17)	0.40006 (12)	0.0183 (4)	
C2	0.64685 (19)	0.81069 (18)	0.35944 (13)	0.0224 (4)	
H2A	0.5944	0.7653	0.3790	0.027*	
C3	0.59018 (19)	0.88051 (19)	0.29053 (12)	0.0234 (4)	
H3A	0.5008	0.8824	0.2651	0.028*	
C4	0.66768 (19)	0.94822 (18)	0.25918 (12)	0.0204 (4)	
C5	0.80152 (19)	0.94533 (17)	0.29981 (11)	0.0192 (4)	
H5A	0.8542	0.9905	0.2802	0.023*	
C6	0.85652 (18)	0.87601 (17)	0.36893 (11)	0.0183 (4)	
H6A	0.9458	0.8754	0.3952	0.022*	
C7	0.84768 (18)	0.81425 (17)	0.58127 (12)	0.0178 (4)	
C8	0.8544 (2)	0.77177 (19)	0.65816 (13)	0.0262 (5)	
H8A	0.8605	0.6918	0.6680	0.031*	
C9	0.8520 (2)	0.8473 (2)	0.71996 (13)	0.0286 (5)	
H9A	0.8567	0.8174	0.7709	0.034*	
C10	0.84255 (19)	0.96751 (18)	0.70672 (12)	0.0204 (4)	
C11	0.83575 (18)	1.01047 (18)	0.63009 (12)	0.0197 (4)	
H11A	0.8293	1.0904	0.6201	0.024*	
C12	0.83859 (18)	0.93414 (17)	0.56862 (12)	0.0186 (4)	
H12A	0.8343	0.9639	0.5178	0.022*	
C13	1.03380 (18)	0.72022 (17)	0.50883 (12)	0.0178 (4)	
C14	1.0843 (2)	0.64556 (18)	0.46419 (12)	0.0214 (4)	
H14A	1.0332	0.5857	0.4330	0.026*	
C15	1.2091 (2)	0.65933 (19)	0.46564 (12)	0.0230 (4)	
H15A	1.2408	0.6092	0.4351	0.028*	
C16	1.28772 (19)	0.74795 (19)	0.51259 (12)	0.0209 (4)	
C17	1.24043 (19)	0.81979 (18)	0.56012 (12)	0.0198 (4)	
H17A	1.2931	0.8768	0.5938	0.024*	
C18	1.11392 (19)	0.80605 (17)	0.55710 (11)	0.0190 (4)	
H18A	1.0825	0.8555	0.5881	0.023*	
C19	0.4396 (2)	1.0054 (2)	0.12929 (14)	0.0353 (6)	
H19A	0.4018	1.0427	0.0765	0.053*	
H19B	0.3984	1.0333	0.1660	0.053*	
H19C	0.4281	0.9225	0.1228	0.053*	
C20	0.7909 (3)	1.1960 (2)	0.73922 (14)	0.0367 (6)	
H20A	0.7705	1.2484	0.7761	0.055*	
H20B	0.7164	1.1862	0.6899	0.055*	
H20C	0.8606	1.2276	0.7252	0.055*	
C21	1.5139 (2)	0.8774 (3)	0.58105 (19)	0.0518 (8)	
H21A	1.5994	0.8937	0.5821	0.078*	
H21B	1.5180	0.8531	0.6350	0.078*	
H21C	1.4619	0.9465	0.5652	0.078*	

### Atomic displacement parameters (Å^2^)

**Table:**

	*U*^11^	*U*^22^	*U*^33^	*U*^12^	*U*^13^	*U*^23^
As1	0.01501 (10)	0.01771 (11)	0.02239 (10)	−0.00177 (8)	0.00793 (8)	−0.00007 (8)
S1	0.0216 (3)	0.0365 (3)	0.0284 (3)	−0.0021 (2)	0.0039 (2)	0.0102 (2)
S2	0.0388 (3)	0.0320 (3)	0.0207 (2)	0.0064 (3)	0.0126 (2)	−0.0011 (2)
S3	0.0181 (3)	0.0435 (4)	0.0390 (3)	0.0006 (2)	0.0161 (2)	−0.0026 (3)
C1	0.0159 (9)	0.0198 (10)	0.0199 (9)	−0.0017 (8)	0.0074 (7)	−0.0023 (7)
C2	0.0152 (9)	0.0263 (11)	0.0274 (10)	−0.0050 (8)	0.0096 (8)	0.0010 (8)
C3	0.0136 (9)	0.0296 (12)	0.0265 (10)	−0.0020 (8)	0.0065 (8)	0.0013 (9)
C4	0.0186 (10)	0.0214 (10)	0.0210 (9)	0.0002 (8)	0.0069 (8)	−0.0015 (8)
C5	0.0176 (9)	0.0208 (10)	0.0208 (9)	−0.0029 (8)	0.0088 (7)	−0.0033 (8)
C6	0.0135 (9)	0.0216 (10)	0.0210 (9)	−0.0027 (7)	0.0075 (7)	−0.0029 (8)
C7	0.0122 (9)	0.0209 (10)	0.0220 (9)	0.0005 (7)	0.0081 (7)	0.0017 (7)
C8	0.0351 (13)	0.0199 (11)	0.0282 (11)	0.0051 (9)	0.0168 (9)	0.0061 (8)
C9	0.0379 (13)	0.0277 (12)	0.0230 (10)	0.0047 (10)	0.0143 (9)	0.0071 (9)
C10	0.0164 (9)	0.0260 (11)	0.0199 (9)	0.0016 (8)	0.0077 (7)	0.0003 (8)
C11	0.0166 (9)	0.0191 (10)	0.0243 (9)	0.0007 (8)	0.0085 (8)	0.0010 (8)
C12	0.0175 (9)	0.0197 (10)	0.0210 (9)	−0.0007 (8)	0.0097 (7)	0.0037 (7)
C13	0.0145 (9)	0.0195 (10)	0.0196 (9)	−0.0002 (7)	0.0061 (7)	0.0016 (7)
C14	0.0210 (10)	0.0200 (10)	0.0232 (9)	0.0013 (8)	0.0078 (8)	−0.0015 (8)
C15	0.0215 (10)	0.0256 (11)	0.0239 (10)	0.0044 (9)	0.0106 (8)	−0.0015 (8)
C16	0.0156 (9)	0.0248 (11)	0.0238 (10)	0.0037 (8)	0.0087 (8)	0.0046 (8)
C17	0.0137 (9)	0.0239 (11)	0.0215 (9)	0.0006 (7)	0.0059 (7)	0.0019 (8)
C18	0.0169 (9)	0.0215 (10)	0.0194 (9)	0.0011 (8)	0.0076 (7)	0.0001 (7)
C19	0.0242 (12)	0.0306 (13)	0.0392 (13)	0.0000 (10)	−0.0033 (10)	0.0034 (10)
C20	0.0505 (16)	0.0318 (14)	0.0249 (11)	0.0106 (11)	0.0100 (11)	−0.0038 (9)
C21	0.0193 (12)	0.069 (2)	0.0692 (19)	−0.0159 (13)	0.0189 (12)	−0.0260 (16)

### Geometric parameters (Å, °)

**Table:**

As1—C7	1.9574 (19)	C9—H9A	0.9300
As1—C13	1.960 (2)	C10—C11	1.392 (3)
As1—C1	1.9660 (19)	C11—C12	1.387 (3)
S1—C4	1.760 (2)	C11—H11A	0.9300
S1—C19	1.793 (2)	C12—H12A	0.9300
S2—C10	1.768 (2)	C13—C18	1.388 (3)
S2—C20	1.795 (2)	C13—C14	1.397 (3)
S3—C16	1.765 (2)	C14—C15	1.384 (3)
S3—C21	1.793 (3)	C14—H14A	0.9300
C1—C2	1.393 (3)	C15—C16	1.395 (3)
C1—C6	1.401 (3)	C15—H15A	0.9300
C2—C3	1.388 (3)	C16—C17	1.392 (3)
C2—H2A	0.9300	C17—C18	1.393 (3)
C3—C4	1.402 (3)	C17—H17A	0.9300
C3—H3A	0.9300	C18—H18A	0.9300
C4—C5	1.398 (3)	C19—H19A	0.9600
C5—C6	1.384 (3)	C19—H19B	0.9600
C5—H5A	0.9300	C19—H19C	0.9600
C6—H6A	0.9300	C20—H20A	0.9600
C7—C12	1.389 (3)	C20—H20B	0.9600
C7—C8	1.395 (3)	C20—H20C	0.9600
C8—C9	1.384 (3)	C21—H21A	0.9600
C8—H8A	0.9300	C21—H21B	0.9600
C9—C10	1.394 (3)	C21—H21C	0.9600
			
C7—As1—C13	98.73 (8)	C11—C12—C7	121.50 (18)
C7—As1—C1	97.87 (8)	C11—C12—H12A	119.2
C13—As1—C1	97.15 (8)	C7—C12—H12A	119.2
C4—S1—C19	103.97 (10)	C18—C13—C14	118.11 (18)
C10—S2—C20	102.53 (10)	C18—C13—As1	123.30 (15)
C16—S3—C21	103.15 (11)	C14—C13—As1	118.53 (15)
C2—C1—C6	117.67 (18)	C15—C14—C13	121.01 (19)
C2—C1—As1	118.95 (14)	C15—C14—H14A	119.5
C6—C1—As1	123.38 (14)	C13—C14—H14A	119.5
C3—C2—C1	121.84 (18)	C14—C15—C16	120.41 (19)
C3—C2—H2A	119.1	C14—C15—H15A	119.8
C1—C2—H2A	119.1	C16—C15—H15A	119.8
C2—C3—C4	120.00 (19)	C17—C16—C15	119.12 (18)
C2—C3—H3A	120.0	C17—C16—S3	123.24 (16)
C4—C3—H3A	120.0	C15—C16—S3	117.64 (16)
C5—C4—C3	118.59 (18)	C16—C17—C18	119.79 (19)
C5—C4—S1	116.77 (15)	C16—C17—H17A	120.1
C3—C4—S1	124.64 (15)	C18—C17—H17A	120.1
C6—C5—C4	120.71 (18)	C13—C18—C17	121.48 (19)
C6—C5—H5A	119.6	C13—C18—H18A	119.3
C4—C5—H5A	119.6	C17—C18—H18A	119.3
C5—C6—C1	121.18 (18)	S1—C19—H19A	109.5
C5—C6—H6A	119.4	S1—C19—H19B	109.5
C1—C6—H6A	119.4	H19A—C19—H19B	109.5
C12—C7—C8	118.15 (18)	S1—C19—H19C	109.5
C12—C7—As1	122.86 (14)	H19A—C19—H19C	109.5
C8—C7—As1	118.99 (15)	H19B—C19—H19C	109.5
C9—C8—C7	120.8 (2)	S2—C20—H20A	109.5
C9—C8—H8A	119.6	S2—C20—H20B	109.5
C7—C8—H8A	119.6	H20A—C20—H20B	109.5
C8—C9—C10	120.74 (19)	S2—C20—H20C	109.5
C8—C9—H9A	119.6	H20A—C20—H20C	109.5
C10—C9—H9A	119.6	H20B—C20—H20C	109.5
C11—C10—C9	118.78 (18)	S3—C21—H21A	109.5
C11—C10—S2	123.63 (16)	S3—C21—H21B	109.5
C9—C10—S2	117.58 (15)	H21A—C21—H21B	109.5
C12—C11—C10	120.06 (19)	S3—C21—H21C	109.5
C12—C11—H11A	120.0	H21A—C21—H21C	109.5
C10—C11—H11A	120.0	H21B—C21—H21C	109.5
			
C7—As1—C1—C2	−89.18 (16)	C8—C9—C10—S2	179.13 (18)
C13—As1—C1—C2	170.93 (16)	C20—S2—C10—C11	11.2 (2)
C7—As1—C1—C6	91.80 (17)	C20—S2—C10—C9	−167.94 (19)
C13—As1—C1—C6	−8.09 (17)	C9—C10—C11—C12	−0.1 (3)
C6—C1—C2—C3	−0.1 (3)	S2—C10—C11—C12	−179.27 (15)
As1—C1—C2—C3	−179.13 (16)	C10—C11—C12—C7	0.2 (3)
C1—C2—C3—C4	1.0 (3)	C8—C7—C12—C11	−0.2 (3)
C2—C3—C4—C5	−1.3 (3)	As1—C7—C12—C11	179.94 (14)
C2—C3—C4—S1	179.00 (16)	C7—As1—C13—C18	−9.49 (18)
C19—S1—C4—C5	172.71 (16)	C1—As1—C13—C18	89.65 (17)
C19—S1—C4—C3	−7.6 (2)	C7—As1—C13—C14	173.47 (16)
C3—C4—C5—C6	0.8 (3)	C1—As1—C13—C14	−87.39 (16)
S1—C4—C5—C6	−179.54 (15)	C18—C13—C14—C15	−2.3 (3)
C4—C5—C6—C1	0.2 (3)	As1—C13—C14—C15	174.91 (15)
C2—C1—C6—C5	−0.5 (3)	C13—C14—C15—C16	0.6 (3)
As1—C1—C6—C5	178.50 (14)	C14—C15—C16—C17	2.2 (3)
C13—As1—C7—C12	79.74 (17)	C14—C15—C16—S3	−177.57 (16)
C1—As1—C7—C12	−18.79 (18)	C21—S3—C16—C17	4.1 (2)
C13—As1—C7—C8	−100.12 (17)	C21—S3—C16—C15	−176.15 (18)
C1—As1—C7—C8	161.35 (16)	C15—C16—C17—C18	−3.1 (3)
C12—C7—C8—C9	0.0 (3)	S3—C16—C17—C18	176.61 (15)
As1—C7—C8—C9	179.87 (17)	C14—C13—C18—C17	1.3 (3)
C7—C8—C9—C10	0.1 (3)	As1—C13—C18—C17	−175.74 (14)
C8—C9—C10—C11	−0.1 (3)	C16—C17—C18—C13	1.4 (3)

### Hydrogen-bond geometry (Å, °)

**Table:**

Cg1 is the centroid of the C7–C12 benzene ring.

**Table:**

*D*—H···*A*	*D*—H	H···*A*	*D*···*A*	*D*—H···*A*
C21—H21A···Cg1^i^	0.96	2.55	3.441 (3)	155

Symmetry codes: (i) *x*+1, *y*, *z*.
